# Role of Inhalational Aztreonam Lysine in Lower Airway Infections in Cystic Fibrosis: An Updated Literature Review

**DOI:** 10.7759/cureus.30833

**Published:** 2022-10-29

**Authors:** Mehwish Zeb, Sujan Poudel, Sai Dheeraj Gutlapalli, Ijeoma A Toulassi, Varshitha Kondapaneni, Ivan Cancarevic

**Affiliations:** 1 Internal Medicine, California Institute of Behavioral Neurosciences and Psychology, Fairfield, USA; 2 Family Medicine, California Institute of Behavioral Neurosciences and Psychology, Fairfiled, USA; 3 Psychiatry and Behavioral Sciences, California Institute of Behavioral Neurosciences and Psychology, Fairfiled, USA; 4 Internal Medicine, California Institute of Behavioral Neurosciences and Psychology, Fairfiled, USA

**Keywords:** cystic fibrosis, pseudomonas aeruginosa, aztreonam lysine

## Abstract

Cystic fibrosis (CF) is an inherited disorder most prevalent in the Caucasian population, characterized by a functional abnormality of the transmembrane conductance regulator protein that leads to a wide array of complications, including chronic lung infections. *Pseudomonas aeruginosa* (PA) is a frequently acquired microbe in CF patients and is associated with deterioration in pulmonary function and increased mortality. Inhaled anti-infective agents are an established curative therapy for CF airway infections, especially with chronic PA lung disease. Amongst them, aztreonam lysine for inhalation (AZLI) is an aerosolized monobactam antibiotic aztreonam, approved for use in CF patients nearly a decade ago. This literature review aims to explore studies based on the efficacy, safety, and tolerability of AZLI use in CF patients with pulmonary infections. We searched for all the relevant articles present in PubMed, Google Scholar, Cochrane Library, EMBASE, ClinicalTrials.gov, and Journal of Cystic Fibrosis for our data collection from 2000 to 2020. The use of AZLI has substantially improved lung function, respiratory symptoms, and remarkably reduced sputum PA density in CF patients, thereby improving the patient's overall quality of life. The adverse effects reported were compatible with CF lung disease. Hence, inhalational therapy with AZLI is highly efficacious and safe in the management of chronic airway infections. More clinical trials need to be conducted in the future to assess its long-term clinical benefits and adverse events as well as to explore the role of AZLI in the setting of acute lung infections.

## Introduction and background

Cystic fibrosis (CF) is the life-limiting autosomal recessive inherited condition most prevalent in the Caucasian population, with an incidence of 1 in 2000 to 3000 live births [1]. In the United States, the median predicted survival for CF patients is 47.4 years (95% CI, 44.2-50.3) for those who were born in 2018, according to the CF Foundation 2018 Registry Report [1]. CF is caused by the abnormal transport of chloride ions, resulting in thick, viscous mucus production within the lungs, which leads to impairment of pulmonary function [1].

In patients with CF, lower airway infections continue to be a leading cause of increased morbidity and mortality [2]. *Pseudomonas aeruginosa* (PA) is a frequently encountered pathogen affecting CF airways, leading to steeper deterioration in lung function [3]. Early acquisition of PA infections is with nonmucoid environmental strains that show a good response to antibiotic treatment [4]. Over time, chronic infections are caused by the mucoid phenotype, which is associated with deterioration in lung function, resistance to antibiotic treatment, and worsening quality of life [5,6]. Several treatment options are used to eradicate the pathogen. Amongst them, inhaled anti-infective agents are used to mitigate early and chronic PA infections [5,6].

Inhalational antibiotics are the standard of care in the treatment of chronic *P. aeruginosa* infections because of their minimal systemic effects, good tolerability, and minimal drug-to-drug interaction [7]. Aztreonam lysine for inhalation (AZLI) is a novel aerosolized lyophilized formulation of the monobactam antibiotic aztreonam and has antipseudomonal activity, whereas lysine salt prevents airway inflammation [7]. The US Food and Drug Association (FDA) approved this formulation for use in cystic fibrosis patients in 2010 [8-10]. In clinical practice, we advise AZLI in CF patients who cannot tolerate inhaled tobramycin, or have worsening lung function, during gestation when aminoglycosides are relatively contraindicated and by the patient's choice [11]. It is given through a nebulizer at a dosage of 75 mg inhalation thrice a day, cycling between 28 days on and 28 days off the drug [11].

Several clinical trials were conducted in both children and adults to assess the safety, efficacy, and susceptibility of AZLI to PA infection [12]. Research studies are currently under process to evaluate the role of AZLI in early PA infection, its recurrence after eradication, safety, and efficacy of AZLI in infancy and acute pulmonary exacerbation [12]. Gilead Sciences, Inc. (Foster City, CA) is conducting a phase 3, randomized, double-blind clinical trial to evaluate the efficacy and safety of AZLI among pediatric CF patients aged three months to eighteen years with new-onset PA infection [12]. This study enrolled 149 subjects and started in November 2017 with an anticipated completion time of 2021 [12]. The primary endpoint of the study was to calculate the number of subjects with PA-negative culture after treatment. The secondary endpoint was to calculate the time to PA recurrence (in weeks) after treatment with AZLI [12].

The focus of this review article is to assess the efficacy, safety, and tolerability of AZLI, to highlight its impact on health-related quality of life (HRQoL), and to provide our readers and healthcare professionals in general with an updated review. We used PubMed, Google Scholar, EMBASE, Cochrane library, ClinicalTrials.gov, and the Official Journal of the European Cystic Fibrosis Society, i.e., Journal of Cystic Fibrosis, for our data collection based on MeSH strategy, regular keywords, and combined keywords. We included studies from the last 20 years, either abstracts or full-text articles, published in English. We manually screened articles in the category of clinical trials and observational studies based on their relevancy and detail.

## Review

Quality of life

The Cystic Fibrosis Questionnaire-Revised (CFQ-R) is a disease-specific, validated measure of health-related quality of life (HRQoL) for CF patients, which has several different domains depending upon age [13]. Several studies have shown favorable outcomes from AZLI use. A phase 3, randomized, placebo-controlled trial (AIR-CF1), was performed on 164 subjects who were treated with 75 mg of AZLI thrice a day for 28 days or placebo [7]. The adjusted mean CFQ-R respiratory scores improved for AZLI-treated subjects than for placebo (difference for 28 days, 9.7 points; p<0.001) [7]. The study also found superior changes for 6/11 non-respiratory CFQ-R scores [7].

In an 18-month open-label study, Oermann et al., reported greater improvement on the CFQ-R Respiratory Symptom scale in 274 patients with each course and received up to nine courses of AZLI 75 mg (28 days on/28 days off) twice or thrice a day [14]. In a recent study, Frost et al. designed an open-label randomized cross-over study in 16 participants with CF [15]. In this pilot study, subjects were given AZLI for 14 days along with intravenous colistin (AZLI+IV) or standard dual intravenous therapy (IV+IV) during pulmonary exacerbations requiring hospitalization and showed a change in CFQ-R Respiratory Domain in participants treated with AZLI+IV (83.3% vs. 43.8%, p=0.03) [15]. This report on the promising role of AZLI during acute pulmonary exacerbation showed a new pathway, and more studies are warranted for its application [15]. In 2008, McCoy et al. published a phase 3 study (AIR-CF2), which was carried out between 2005 and 2006 in 56 CF centers in the United States [16]. In this AIR-CF2 trial, 211 patients were randomly treated with AZLI/placebo for 28 days (twice or thrice a day), and CFQ-R respiratory scores were estimated as secondary endpoints [16]. Mean CFQ-R respiratory scores showed a notable improvement (5.01 points, P = 0.02) [16]. The studies related to favorable outcomes on quality of life are shown in Table 1.

**Table 1:** Studies related to favorable outcome on quality of life.

Study Detail	Place of Study	Age Enrolled	Sample Size(n)	Duration of Study	Study Design
Retsch-Bogart et al. [7]	Australia, Canada, New Zealand, and United States	More than six years	164	2005–2007	Phase 3 clinical trial
Oermann et al. [14]	Australia, Canada, New Zealand, and United States	More than six years	274	2005–2008	Phase 3 clinical trial
Frost et al. [15]	United Kingdom	16 years to 65 years	16	2017–2019	Phase 3 clinical trial
McCoy et al. [16]	United States	More than six years	211	2005–2006	Phase 3 clinical trial

Changes in sputum PA density

Various research trials demonstrated a significant level of suppression in sputum PA density with AZLI use [7,14,16,17]. Retsch-Bogart et al. performed a phase 3 clinical trial (AIR-CF1) and showed suppression in sputum PA density (1.45 log10 CFU/g; p<0.001) [7]. In an 18-month open-label project, Oermann et al. observed an improvement in sputum PA density after nine "28" day cycles of AZLI [14]. Frost et al. observed no remarkable change in sputum bacterial density in CF patients between two treatment groups, i.e. (AZLI + IV) and (IV + IV) during acute pulmonary exacerbations [15]. In addition, PA bacterial resistance was enhanced after treatment with double intravenous therapy (IV+IV) compared to AZLI plus intravenous antibiotic (AZLI+IV) [15]. McCoy et al. demonstrated a remarkable reduction in adjusted mean PA sputum density of −0.66 log10 PA CFU/g sputum in the AZLI-treated patients versus placebo on day 28 (95% CI: −1.13 to −1.19; P=0.006) [16]. Wainwright et al. performed a randomized, placebo-controlled trial between 2008 and 2009 in multiple CF centers [17]. After applying inclusion and exclusion criteria, 157 patients were treated with either AZLI (75 mg) or placebo thrice a day, and subjects were monitored on day 14, day 28 (completion of treatment), and day 42 [17]. One of the secondary endpoints was a change in sputum PA density from the baseline, which was assessed on day 28 from the baseline [17]. This trial yielded statistically significant change for adjusted mean log10 sputum PA colony-forming units (−1.2 log10 CFU/g; AZLI = −1.4; placebo = −0.14; p=0.016), thereby favoring AZLI [17]. 

In 2009, McCoy et al. performed a phase 3 trial on AZLI in CF patients with drug-resistant PA (DRPA) infection and demonstrated a reduction in sputum PA density [18]. Tiddens et al. designed a phase 2, single-arm ALPINE clinical trial from 2011 to 2013, to assess the role of AZLI in suppressing new-onset PA infection among pediatric patients (age three months to eighteen years) [19]. This study depicted that 89.1% of patients were free of PA infection after 28 days and 58.2% remained culture-negative throughout the follow-up visits after the completion of treatment [19]. The studies related to changes in sputum PA density are shown in Table 2.

**Table 2:** Studies related to changes in sputum PA density. CF: cystic fibrosis; PA: *Pseudomonas aeruginosa*.

Study detail	Age enrolled	Duration of study	Sample size (n)	Study design	Result
Oermann et al. [14]	More than six years	2005–2008	274	Phase 3 clinical trial	Trial showed improvement in sputum PA density
McCoy et al. [16]	More than six years	2005–2006	211	Phase 3 clinical trial	Trial yielded notable suppression of sputum PA density (p=0.006)
Wainwright et al. [17]	six years and older	2008–2009	157	Phase 3 clinical trial	Study showed a notable reduction in sputum PA density (p=0.016)
Tiddens et al. [19]	three month to 18 years	2011–2013	105	Phase 2 clinical trial	This trial showed a reduction in sputum PA density in 89.1% of CF patients

Changes in FEV1 from baseline

Recurrent airway infections have detrimental effects on lung function, but the introduction of AZLI has addressed this issue and improved outcomes effectively, as evident from several clinical trials and observational studies [7,14,15,17]. Retsch-Bogart et al. demonstrated remarkable improvement in respiratory symptoms in patients with moderate to severe lung diseases at baseline and no prior use of anti-pseudomonal antibiotics [7]. On completion of treatment, the mean adjusted forced expiratory volume in one second (FEV1) increased (95% CI, 6.3 to 14.3; p < 0.001) and remained above baseline after treatment [7]. In 2010, Oermann et al. published a clinical trial that showed a sustained improvement in FEV1 from baseline over nine on/off cycles of AZLI [14]. In a recent phase 4 study published in 2019, Frost et al. conducted a randomized, controlled cross-over, pilot study to assess the role of AZLI during acute pulmonary exacerbations compared to standard intravenous therapy [15]. Sixteen subjects were randomized between two treatment groups: AZLI with an intravenous antibiotic, i.e., colistin (AZLI+IV), and standard double intravenous therapy (IV+IV) [15]. This trial revealed significant changes in FEV1 from baseline in the AZLI+IV treatment group compared to double IV therapy (IV+IV) (p=0.002) [15]. In a phase 3 clinical trial, McCoy et al. showed the adjusted mean difference for percent predicted FEV1 (ppFEV1) between AZLI and the placebo-treated group was 6.3% (p=0.001) [16].

In a phase 3b (AIR-CF4) clinical trial, Wainwright et al. also observed a mean relative change in FEV1% predicted at 2.7% (p=0.021) in a 12-month clinical trial [17]. Moreover, AZLI showed promising results in patients with baseline FEV1 less than 90% predicted (p=0.032) in comparison to FEV1 more than 90% predicted (p=0.302) [17]. In a retrospective cohort study published in 2017, Morton et al. assembled study data from two years before (t-2) and after (t+2) starting AZLI and depicted changes in FEV1 were arrested with continual treatment [20]. Plant et al. designed a study on inhaled aztreonam lysine (AZLI), and data were collected from multiple centers in Ireland and the UK [21]. This trial divided 24 months into four time zones of six-month intervals each [21]: zero to six months (P1), six to twelve months (P2) prior to initiation of AZLI and zero to six months (T1), six to twelve months (T2) after starting AZLI, and FEV1 was monitored across all time zones [21]. After analyzing the data, it was revealed that the median FEV1 predicted had worsened from P2 to P1 (p=0.001) but improved notably from P1 to T1 after the initiation of AZLI (p=0.03) [21].

Flume et al. performed a comparative trial in multiple centers in the US for 28 weeks and exhibited improvement in the adjusted mean of FEV1 in AZLI-treated groups (1.37[0.67]) from baseline compared to placebo (0.04[0.66]), though the difference between the two groups was not remarkable statistically (95% CI, 1.33 [−0.55, 3.20] p=0.16) [22]. The articles related to changes in FEV1 from baseline are shown in Table 3.

**Table 3:** Articles related to changes in FEV1 from baseline. FEV1: forced expiratory volume in one second; AZLI: aztreonam lysine for inhalation.

Study detail	Duration of study	Study design	Age enrolled	Result
Oermann et al. [14]	2005–2008	Phase 3 clinical trial	More than six years	Sustained improvement in FEV1 from baseline over nine cycles of AZLI
Frost et al. [15]	2017–2019	Phase 4 clinical trial	16 years to 65 years	P=0.002
Wainwright et al. [17]	2008–2009	Phase 3 clinical trial	Six years and older	Baseline FEV1 <90% predicted (p=0.032) baseline FEV1 >90% predicted (p=0.302)
Plant et al. [21]	2012–2014	Retrospective and prospective study	18 years and older	p=0.001
Montgomery et al. [23]	2012–2015	Phase 3 clinical trial	Six years and older	P=0.16

Safety and tolerability

AZLI is a safe and well-tolerated inhaled anti-infective agent. Various studies worked on the safety profile of AZLI and found that it has adverse effects as well, apart from its clinical efficacy [7,14,16,17]. Retsch-Bogart et al. monitored the safety and tolerability of AZLI in a phase 3 clinical trial [7]. The study revealed a productive cough in the AZLI-treated group compared to the placebo (p=0.047) [7]. The reactive airway was observed in three patients and five patients for AZLI and placebo-treated groups, respectively [7]. No notable changes were found in the laboratory profile and vitals, and also, mortality or anaphylactic reactions were not reported [7]. In an 18-month phase 3 clinical trial (AIR-CF3), Oermann et al. also monitored the long-term safety profile of AZLI, including adverse effects, CF aggravating respiratory and non-respiratory symptoms, laboratory levels, and vitals of patients [14]. The most frequent adverse effect was attributed to respiratory symptoms (cough 89.4%; productive cough 80.3%) [14]. Serious adverse effects of AZLI were 44.7% for the twice-a-day group and 52.4% for the thrice-a-day group [14]. Non-respiratory symptoms were anorexia, fever, fatigue, and headache [14]. Moreover, no remarkable changes were observed in vitals and laboratory variables [14]. McCoy et al. also assessed the safety of AZLI in a randomized, placebo-controlled trial in 211 subjects in multiple CF centers [16]. The study observed that adverse events were not statistically remarkable in all three treatment groups (AZLI twice-daily dosage BID; thrice daily dosage TID; and placebo) [16]. Out of 211 subjects, nine needed hospitalization (seven with pulmonary exacerbation, one with hyponatremia, and one with intestinal obstruction) [16]. Hence, AZLI-treated groups were well-tolerated and adverse effects were compatible with CF respiratory symptoms [16]. Wainwright et al. also demonstrated that cough was the most common adverse event observed in the AZLI-treated group (35[46%]) compared to the placebo (35 [38%]) [17]. The studies showing the safety and tolerability of AZLI are shown in Table 4.

**Table 4:** Studies showing safety and tolerability of AZLI. AZLI: aztreonam lysine for inhalation.

Study detail	Duration of study	Age enrolled	Sample size (n)	Study design	Result
Retsch-Bogart et al. [7]	2005–2007	More than six years	164	Phase 3 clinical trial (AIR-CF1)	Trial showed that adverse events were compatible with respiratory symptoms. There was no remarkable change in vitals and laboratory profile and no death was observed.
Oermann et al. [14]	2005–2008	More than six years	274	Phase 3 clinical trial	Most frequent adverse effect observed was respiratory symptoms.
McCoy et al. [16]	2005–2006	More than six years	211	Phase 3 clinical trial (AIR-CF2)	Study yielded no remarkable adverse effects in all treatment groups. Only nine patients were hospitalized (seven from pulmonary exacerbation, one with hyponatremia, one with intestinal obstruction).
Wainwright et al. [17]	2008–2009	Six years and older	157	Phase 3 clinical trial	This study showed that cough was the most common adverse effect observed in AZLI-treated group compared to placebo.

Hospitalization rates

Retsch-Bogart et al. also demonstrated that hospitalization had lessened in the AZLI-treated group compared to the placebo (5% versus 14%), though it was not statistically remarkable (p = 0.064) [7]. In the AIR-CF3 clinical trial, Oermann et al. showed a reduction in hospitalization in CF patients with AZLI use [14]. The most frequent cause of hospitalization is the emergence of lower respiratory tract infections [14]. The total hospitalization rate per patient year was 0.897, while the total respiratory hospitalization rate per patient year was 0.793 [14]. McCoy et al. monitored hospitalization data from admission to discharge using an electronic case report form from day zero to day 84 [16]. It showed no significant difference in time to the first hospitalization in CF patients between the AZLI-treated group and the placebo group [16]. The mean number of hospitalization days was 0.5 (placebo-treated group) and 0.9 (AZLI-treated group), respectively [16].

Plant et al. also showed a significant change in hospitalization after the initiation of AZLI [21]. Hospital bed days were reduced from 15.0 to 12.6 (p=0.05) [21]. In a retrospective case-control study (AIR-CF3), Montgomery et al. revealed a notable reduction in hospitalization risk in CF patients with AZLI use compared to standard-of-care (SOC) therapy (p=0.020) [23]. The rate of hospitalization per patient year was 0.91 for the AZLI group and 1.26 for the SOC therapy group [23]. The AZLI-treated group had a 28% lower risk of hospitalization compared to SOC [23]. Studies related to hospitalization rates are shown in Table 5.

**Table 5:** Studies related to hospitalization rates.

Study detail	Duration of study	Age enrolled	Sample size(n)	Study design
Retsch-Bogart et al. [7]	2005–2007	More than six years	164	Phase 3 clinical trial
Oermann et al. [14]	2005–2008	More than six years	274	Phase 3 clinical trial
McCoy et al. [16]	2005–2006	More than six years	211	Phase 3 clinical trial
Plant et al. [21]	2012–2014	18 years and older	117	Retrospective and prospective study

AZLI in pediatric CF patients

Various phase 3 and phase 4 clinical trials demonstrated the efficacy and safety of AZLI among pediatric CF patients, which are discussed below [7,14,16,17,19,24]. Studies showed an increase in the percent predicted in FEV1 in children over six years [7,16]. Also, CF patients experienced an improvement in CFQ-R RSS scores, a reduction in sputum PA density, and fewer respiratory adverse events were observed [7,16]. Wainwright et al. also demonstrated improvement in FEV1 from baseline [17]. Tiddens et al. conducted an open-label phase 2 clinical trial in pediatric patients aged three months to eighteen years [19]. It showed that 89.1% of CF patients were PA(-) after four weeks of AZLI therapy and 75.2% after eight weeks [19]. Clinical trials conducted in pediatric CF patients are shown in Table 6.

**Table 6:** Clinical trials conducted in pediatrics CF patients. FEV1: forced expiratory volume in one second; CF: cystic fibrosis; ppFEV1: percent predicted forced expiratory volume in one second; PA: *Pseudomonas aeruginosa*; CFQ-R: Cystic Fibrosis Questionnaire-Revised.

Study detail	Duration of study	Study design	Age enrolled	Result
Retsch-Bogart et al. [7]	2005–2007	Phase 3 clinical trial	More than six years	Study demonstrated an increase in FEV1.
Oermann et al. [14]	2005–2008	Phase 3 clinical trial	More than six years	Showed reduction in hospitalization rates and sputum PA density
McCoy et al. [16]	2005–2006	Phase 3 clinical trial	More than six years	CF patients experienced an improvement in ppFEV1 and CFQ-R scores
Wainwright et al. [17]	2008–2009	Phase 3 clinical trial	Six years and older	Trial showed improvement in FEV1 and reduction in sputum PA density
Tiddens et al. [19]	2011–2013	Phase 2 clinical trial	Three months to 18 years	Study demonstrated 89.1% were PA(-) respiratory cultures after 28 days of treatment
ClinicalTrials.gov [24]	2011–2013	Phase 3 clinical trial	Less than 12 years	Subjects showed improvement in CFQ-R scores and FEV1

## Conclusions

This review signifies that AZLI is an effective and well-tolerated inhalational anti-infective agent in CF patients with chronic PA infections. Cyclic therapy of AZLI (28 days on/28 days off) has a promising role in preserving lung function, reducing bacterial density in sputum, improving patients' life expectancy, and reducing hospitalisation rate, thereby enhancing the health quality of CF patients. Despite all the progress, more studies are warranted for this therapy to find out the long-term adverse effects, its clinical benefits in the long run with a larger sample size and extensive duration of follow-ups, and its safety as well as efficacy in infancy. It is also necessary to monitor the emergence of PA resistance over time to prevent eventual reinfection.
